# Arabidopsis NPF4.6 and NPF5.1 Control Leaf Stomatal Aperture by Regulating Abscisic Acid Transport

**DOI:** 10.3390/genes12060885

**Published:** 2021-06-08

**Authors:** Takafumi Shimizu, Yuri Kanno, Hiromi Suzuki, Shunsuke Watanabe, Mitsunori Seo

**Affiliations:** 1 RIKEN Center for Sustainable Resource Science, Kanagawa 230-0045, Japan; takshim@bs.naist.jp (T.S.); yuri.kanno@riken.jp (Y.K.); hiromi.suzuki.rg@riken.jp (H.S.); shunsuke.watanabe@riken.jp (S.W.); 2 Graduate School of Science and Technology, Nara Institute of Science and Technology, Nara 630-0192, Japan

**Keywords:** abscisic acid (ABA), *Arabidopsis thaliana* L., guard cells, NITRATE TRANSPORTER 1/PEPTIDE TRANSPORTER FAMILY (NPF), transporter

## Abstract

The plant hormone abscisic acid (ABA) is actively synthesized in vascular tissues and transported to guard cells to promote stomatal closure. Although several transmembrane ABA transporters have been identified, how the movement of ABA within plants is regulated is not fully understood. In this study, we determined that Arabidopsis NPF4.6, previously identified as an ABA transporter expressed in vascular tissues, is also present in guard cells and positively regulates stomatal closure in leaves. We also found that mutants defective in NPF5.1 had a higher leaf surface temperature compared to the wild type. Additionally, NPF5.1 mediated cellular ABA uptake when expressed in a heterologous yeast system. Promoter activities of *NPF5.1* were detected in several leaf cell types. Taken together, these observations indicate that NPF5.1 negatively regulates stomatal closure by regulating the amount of ABA that can be transported from vascular tissues to guard cells.

## 1. Introduction

The plant hormone abscisic acid (ABA) plays crucial roles in various processes such as seed development, germination and responses to abiotic and biotic stresses [1,2,3]. Frequently, these physiological responses are associated with changes in endogenous ABA levels. For example, ABA levels increase in response to water deficit to induce stomatal closure and the expression of stress-responsive genes [4,5,6]. In developing seeds, ABA levels peak during the middle stage when seeds accumulate storage compounds and acquire desiccation tolerance [7,8,9]. Therefore, the concentration of ABA within plants is a key determinant of ABA-mediated physiological responses.

The amount of ABA within a cell is defined by the balance between biosynthesis and catabolism. To date, most of the genes and enzymes involved in ABA metabolism have been identified [10]. It is now widely accepted that the enzymatic reaction catalyzed by nine-*cis*-epoxycarotenoid dioxygenase (NCED) is the rate-limiting step for ABA biosynthesis. Among five NCEDs in Arabidopsis (NCED2, 3, 5, 6 and 9), NCED3 plays a central role in the drought-inducible accumulation of ABA in vegetative organs [11,12]. On the other hand, NCED6 and NCED9 are the main NCEDs that contribute to ABA production in Arabidopsis seeds [13]. Expression of *NCED3* is rapidly induced upon water deficit [11,14], whereas mRNA levels of *NCED6* and *NCED9* are regulated by environmental factors that affect seed dormancy and germination (e.g., light and temperature) during imbibition [15,16]. Recently, the expression of *NCED3* was reported to be regulated by the CLE25 peptide [17]. A transcription factor (NGATHA1) that directly binds to the *NCED3* promoter has also been identified [18], although its relationship to CLE25 is currently unknown. A number of studies in other plant species have documented the presence of NCED(s) in vegetative organs and seeds whose expression is regulated by environmental factors [4,19,20,21,22,23]. ABA 8’-hydroxylase, belonging to the CYP707A subfamily of cytochrome P450s, is well established as the rate-limiting enzyme of ABA catabolism. There are four CYP707As (CYP707A1-4) that function as ABA 8’-hydroxylases in Arabidopsis [24,25] and differentially regulate various physiological processes. For example, the expression of *CYP707A3* is strongly induced by rehydration of plants after dehydration, indicating that the gene product plays an important role in drought stress responses [24,26]. In fact, mutants defective in CYP707A3 accumulate higher levels of ABA during water deficit and subsequent rehydration compared to the wild type. In contrast, *CYP707A1* and *CYP707A2* are highly expressed in the middle and late stages of seed development, respectively. Mutant seeds defective in these genes exhibit deeper dormancy and have a higher ABA content compared to the wild type [27]. The physiological functions of CYP707As that are involved in ABA catabolism have also been studied in other plant species [22,23,28,29,30].

In addition to the well-studied regulatory roles of ABA biosynthesis and catabolism, recent studies have demonstrated that transmembrane transport of ABA is also an important factor determining the cellular hormone content and, hence, the resulting physiological responses [10,31]. Studies on the localization of ABA biosynthetic enzymes within plants have shown that leaf vascular tissues, more specifically phloem companion cells, are the main site of ABA biosynthesis during water stress responses [5,32,33]. This finding indicates that ABA synthesized in vascular tissues has to be translocated to guard cells to induce stomatal closure. In fact, three types of transporter families, namely ATP binding cassette (ABC), NITRATE TRANSPORTER 1/PEPTIDE TRANSPORTER FAMILY (NPF) and Detoxification Efflux Carriers (DTX)/Multidrug and Toxic Compound Extrusion (MATE) proteins that possibly regulate the process have been identified. In Arabidopsis, genes encoding the subgroup G ABC protein (ABCG) ABCG25 and the NPF protein NPF4.6, which was originally identified as the low-affinity nitrate transporter NRT1.2 and also called AIT1 after ABA IMPORTING TRANSPORTER 1, are expressed in vascular tissues and possibly facilitate ABA export and import from/into the cells, respectively [34,35]. Genes encoding another ABCG protein ABCG40 (also referred to as PDR12 after PLEIOTROPIC DRUG RESISTANCE 12) and the DTX/MATE protein DTX50 that functions as an ABA importer and exporter, respectively, are expressed in guard cells [36,37]. In Arabidopsis seeds, ABCG25 and ABCG31 export ABA from the endosperm, whereas ABCG30 and ABCG40 import ABA into the embryo [38]. Possible ABA transporters have also been identified in several other plant species [10,39,40,41,42,43,44]. These investigations indicate that the movement of ABA within plants must be regulated by multiple steps.

In this study, we found that Arabidopsis NPF4.6 was localized in the guard cells of leaves in addition to vascular tissues. Furthermore, we found that mutants defective in NPF5.1 (*npf5.1*) had higher leaf surface temperatures compared to the wild type. When expressed in yeast cells, NPF5.1 mediated cellular ABA uptake, indicating that this protein also functions as an ABA importer. We discuss how the two NPFs coordinately regulate stomatal aperture through the transport of ABA.

## 2. Materials and Methods

### 2.1. Plant Materials and Growth Conditions

Arabidopsis (*Arabidopsis thaliana* (L.) Heynh.) accession Columbia-0 (Col-0) was used as the wild type. T-DNA insertion lines were obtained from the Arabidopsis Biological Resource Center. *aao3-4* (SALK_072361) and *npf4.6-1* (*ait1-1*) (SALK_146143) were isolated in previous studies [35,45]. Homozygous *npf5.1-1* (SALK_085919) and *npf5.1-2* (SALK_000464) were selected by PCR using primer combinations designed by the T-DNA Primer Design Tool (http://signal.salk.edu/tdnaprimers.2.html, accessed on 25 March 2014). Transgenic plant lines (*pNPF4.6:gNPF4.6-GUS*, *35S:NPF5.1* and *pNPF5.1:GUS*) were generated by transformation using Agrobacterium strain GV3101. 

After surface sterilization with 70% (*v/v*) ethanol and then with 5% (*v/v*) NaClO (0.25% active chlorine) containing 1% (*w/v*) Tween 20, seeds were sown on plates containing half-strength MS media and 0.8% (*w/v*) agar. The plates were incubated at 4 °C for 3 d in the dark and then incubated at 22 °C under continuous light (50 μ moles/m ^2^/s). For measurements of leaf surface temperature and quantification of plant hormones, young seedlings (around 7-d-old) were transplanted on soil containing vermiculite and Metro-mix 350 (Sun Gro Horticulture, MA, USA) at a 3:1 ratio and grown under day (10 h) and night (14 h) conditions. For GUS staining and seed propagation for germination assays, plants were grown under continuous light after transplanting. 

### 2.2. Germination Assays

Surface sterilized seeds were sown on plates containing half-strength MS media and 0.8% (*w/v*) agar with or without supplementation with 0.5 μM ABA. ABA was dissolved in DMSO at a concentration 1000 times greater than the final concentration used in the assay. The stock ABA solution was added to the cooled autoclaved media. DMSO was added to the control media. Plates were incubated at 4 °C for 3 d, and the numbers of germinated seedlings with green cotyledons were scored after transferring the plates to lighted conditions at 22 °C. 

### 2.3. Vector Construction

For expression of NPF5.1 in yeast, *NPF5.1* CDS cloned in pENTR/D-TOPO (Thermo Fisher, Waltham, MA, USA) was transferred to pYES-DEST52 (Thermo Fisher, Waltham, MA, USA) by LR reactions [46].

To generate *pNPF4.6:gNPF.6-GUS* plants, a promoter region (2 kb upstream of the ATG start codon) of *NPF4.6* was amplified by PCR with the primer pair 5′-CACCATTAATATATTGCGGCTA-3′/5′-TCTCTCTCTTTCTTTCTCTC-3′ and cloned into pENTR/D-TOPO. The genomic sequence of *NPF4.6* was amplified by PCR with the primer pair 5′-AAAGAAAGAGAGAGAATGGTGGGTTCTTGAAGCTTCTCACATTATTTTC-3′/5′-GGCGCGCCCACCCTTGCTTCTTGAACCAGTTGATCTATACTTGTA-3′ and combined with the 2 kb upstream region cloned in pENTR/D-TOPO that had been linearized by inverse PCR with the primer pair 5′-AAGGGTGGGCGCGCCGACCCAGC-3′/5′-TCTCTCTCTTTCTTTCTCTCAAACTTTGAG-3′ using an In-Fusion Cloning Kit (Takara Bio, Shiga, Japan). The *NPF4.6* promoter plus genomic sequence was cloned into pGWB3 [47] by LR reactions. 

For guard-cell specific expression of *NPF4.6*, the Arabidopsis *MYB60* promoter region [48] and the *NPF4.6* CDS were amplified by PCR with primer pairs 5′-AAGCTTGGGTTCCCTCTGCTGTATG-3′/5′-GTCGACCTTTCTCTCTCTCTCTTCCTCTAG-3′ and 5′-TCTAGAGTCGACATGGAAGTGGAAGAAGAGGT-3′/5′-GGTACCTTAGCTTCTTGAACCAGTTG-3′, respectively, and cloned into pT7Blue (Novagen, Darmstadt, Germany). The *MYB60* promoter sequence was cloned into pBIB-HYG [49] using the Hind III and Sal I restriction sites. *NPF4.6* CDS was then cloned into pBIB-HYG containing *MYB60* promoter using the *Sal* I and *Kpn* I restriction sites.

For transient expression of GFP-fused NPF5.1 in onion epidermal cells, the *NPF5.1* CDS cloned in pENTR/D-TOPO was transferred to pUGW5 [47] by LR reactions. pUGW2 [47] was used for the transient expression of GFP alone.

To generate *35S:NPF5.1* plants, the *NPF5.1* CDS was amplified by PCR with a primer pair 5′-GCCGCCCCCTTCACCATGGAGGCTGCAAAAGTTTA-3′/5′-AGCTGGGTCGGCGCGTTAGATACTAAGAGGAGATGTGTC-3′ and cloned into pENTR4 (Thermo Fisher) using an In-Fusion Cloning Kit (Takara Bio). After confirming the sequence, the *NPF5.1* CDS was cloned into pGWB402-omega [50] by LR reactions.

To generate *pNPF5.1:GUS* plants, a promoter (the 3 kb region upstream of the ATG start codon) region of *NPF5.1* was amplified by PCR with the primer pair 5′-GCCGCCCCCTTCACCTACTAAGATGTTTATTGGTTGA-3′/5′-AGCTGGGTCGGCGCGTAATTGTTTTTTCTGTTTATATCAA-3′ and cloned into pENTR/D-TOPO that had been linearized by inverse PCR with the primer pair 5′-CGCGCCGACCCAGCTTTCTTG-3′/5′-GGTGAAGGGGGCGGCCGCGG-3′ using an In-Fusion Cloning Kit. The *NPF5.1* promoter sequence was cloned into pGWB3 by LR reactions.

### 2.4. Thermal Imaging

Thermal images were obtained using a thermal video system (TVS-8500; Nippon Avionics, Kanagawa, Japan).

### 2.5. GUS Staining

GUS staining was performed using a solution composed of 50 mM sodium phosphate buffer (pH 7.2), 10 mM EDTA, 0.05% (*v/v*) Triton X-100, 0.5 mM potassium ferrocyanide, 0.5 mM potassium ferricyanide and 1 mM X-Gluc. Close-up views were observed using a Leica M205 stereo microscope or a Leica DM2500 (Leica Camera AG, Wetzlar, Germany) microscope.

### 2.6. Transport Assays

The *NPF5.1* CDS cloned in pYES-DEST52 was introduced into the *Saccharomyces cerevisiae* strain INVSc1 (Thermo Fisher). As a negative control, yeast cells were transformed with the empty pYES-DEST52 vector. Assays were conducted as described previously [51]. Yeast cells expressing *NPF5.1* were incubated with 1 or 10 μM substrates for 10 min in 50 mM potassium phosphate buffer (KPB) (pH 5.8). Extraction, purification, quantification by LC-MS/MS were performed, as described previously [44].

### 2.7. ABA Measurements

Endogenous levels ABA were quantified as described previously [51]. Samples were purified using Oasis HLB, MCX and WAX column cartridges (1 cc, Waters, MA, USA).

### 2.8. Chemicals

(±)-ABA was purchased from Sigma-Aldrich (MO, USA). (+)-ABA was purchased from Tokyo Chemical Industry Co (Tokyo, Japan). (D_6_) ABA was purchased from Icon Isotope (MI, USA).

(±)-ABA was used for germination assays, whereas (+)-ABA was used for transport assays.

### 2.9. Quantitative Reverse Transcription-PCR

Quantification of mRNA levels was performed as described previously [52]. Total RNA was prepared from 7-d-old seedlings of wild type and *npf5.1* mutants. The primer pair 5′-GCGGTTTGGATCACTCCCAT-3′/5′-GTCAAGAGAATCATCCCCAGGAC-3′ was used to detect *NPF5.1* cDNA. Expression levels were normalized against the levels of 18S rRNA.

### 2.10. Transient Expression of GPF Fused NPF5.1 in Onion Epidermal Cells

One milligram of gold particles (1 µm diameter, Bio-Rad, Hercules, CA, USA) were coated with 2 µg plasmid DNA for each bombardment. Onion bulb scales were placed with their inner side up on 1.5% (*w/v*) agar plates. Gold particles coated with plasmid DNA were introduced into the inner epidermal cells of an onion by particle bombardment at 1100 psi using a PDS-1000 He Biolistic Particle Delivery System (Bio-Rad). After bombardment, the onion bulb scales were incubated in moist conditions overnight at 25 °C in the dark. GFP fluorescence was observed using a confocal laser scanning microscope LSM700 (Carl Zeiss, Oberkochen, Germany). Onion epidermal peels were stained with 50 μM propidium iodide solution to visualize the cell walls. To observe plasmolysis, 20% (*w/v*) mannitol was used.

## 3. Results

### 3.1. NPF4.6 Mediates ABA Uptake into Guard Cells

**Figure 1:**
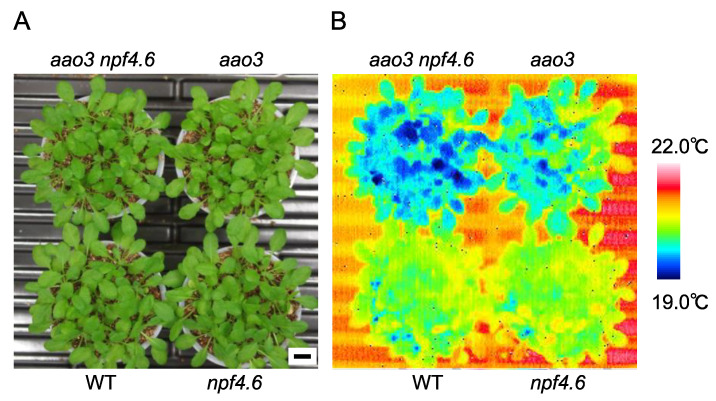
NPF4.6 is a positive regulator of stomatal closure. (**A**) Wild type (WT), *npf4.6-1* (*npf4.6*), *aao3-4* (*aao3*) and *aao3-4 npf4.6-1* (*aao3 npf4.6*) plants (approximately 1-month-old) grown under a day and night cycle (10 h light/14 h dark). Scale bar = 1 cm. (**B**) Leaf surface temperature of plants in (**A**) observed by a thermal imaging camera.

We previously showed that NPF4.6 functions as an ABA importer in Arabidopsis [35]. The inflorescence stems of mutants defective in NPF4.6 (*npf4.6*) had lower surface temperatures with more open stomata; however, the mutants did not show any clear leaf phenotypes, presumably due to redundancies with other ABA transporters. Therefore, the physiological roles of NPF4.6 remained not fully understood. To examine the contribution of NPF4.6 in the regulation of leaf stomatal apertures, we first investigated the effects of reduced endogenous ABA levels on the phenotypes of *npf4.6*. In Arabidopsis, there are four genes encoding aldehyde oxidase (*AAO1*-*4*) [53]. Although the *AAO3* product plays a central role in the conversion of abscisic aldehyde to ABA, other isoforms could replace the function of AAO3, at least in the loss of function *aao3* mutant background [45,53,54]. Therefore, *aao3* has a relatively mild phenotype. When cultivated under well-watered conditions, *aao3* did not show the growth retardation that is a typical symptom of mutants severely affected in ABA biosynthesis (Figure 1); however, the leaf surface temperature of the mutant was reduced compared to the wild type. In the same conditions, *npf4.6* was not distinguished from wild type in terms of leaf surface temperature as we reported previously. Nevertheless, we found that the *npf4.6* mutation was able to enhance the “cool” phenotype observed in the *aao3* mutant background (Figure 1), indicating that NPF4.6 is potentially a positive regulator of stomatal closure.

**Figure 2:**
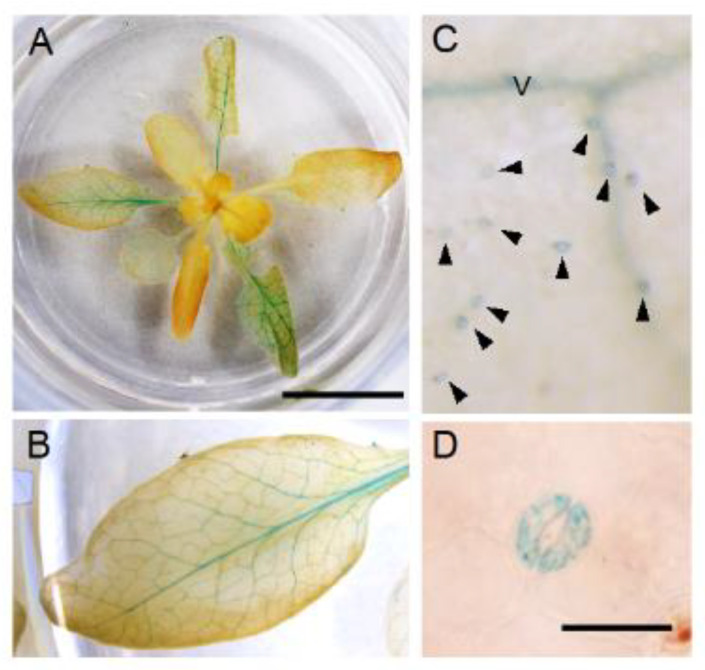
GUS staining of *pNPF4.6:gNPF4.6-GUS* plants. (**A**) A rosette (approximately 3 weeks old). Scale bar = 1 cm. (**B**) Close-up view of a leaf from (**A**). (**C**) Close-up view of the leaf surface. Arrowheads indicate guard cells. V denotes vascular tissues. (**D**) Close-up view of a pair of guard cells. Scale bar = 20 μm. Plants were grown in continuous light.

Since the promoter activities of NPF4.6 were detected in the vascular tissues of leaves where ABA is supposed to be synthesized [35], we hypothesized that the protein regulates the amount of ABA transported from the tissues by mediating cellular ABA uptake. However, if this is the case, one may imagine that the loss of NPF4.6 function would result in an increased amount of ABA transported from the vascular tissues to guard cells and, hence, reduce the stomatal aperture (increased leaf surface temperature). One possible explanation for this unexpected result is that NPF4.6 is present also in guard cells and transports ABA into the cells. To examine this possibility, we generated transgenic plants that contained a transgene in which the *GUS* reporter gene was placed after the *NPF4.6* genomic sequence so that the GUS fused NPF4.6 protein was expressed under the control of the *NPF4.6* native promoter (*pNPF4.6:gNPF4.6-GUS*) (Figure 2). As in the case of simple promoter-GUS lines, GUS staining was clearly detected in leaf vascular tissues in the *pNPF4.6:gNPF4.6-GUS* plants (Figure 2A,B). In addition, closer investigation revealed that guard cells of the transgenic plants were also stained with GUS (Figure 2C,D). These results indicated that NPF4.6 is localized to both the vascular tissues and the guard cells.

**Figure 3:**
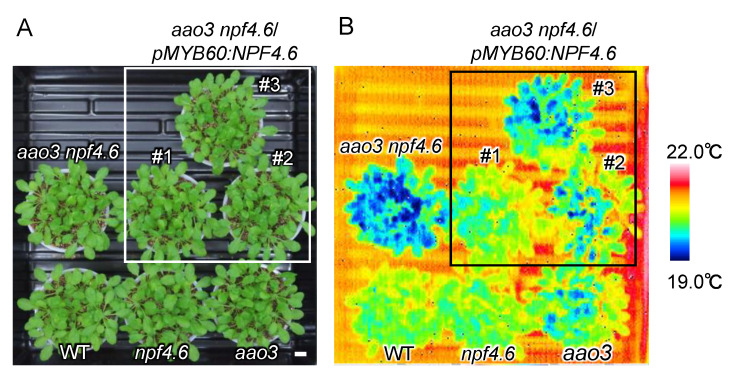
Guard-cell specific expression of *NPF4.6* in the *aao3 npf4.6* double mutant background. (**A**) Wild type (WT), *npf4.6-1* (*npf4.6*), *aao3-4* (*aao3*), *aao3-4 npf4.6-1* (*aao3 npf4.6*) and three independent lines (#1-3) of *aao3-4 npf4.6-1* that express wild-type *NPF4.6* specifically in the guard cells (*aao3 npf4.6*/*pMYB60:NPF4.6*). Plants (approximately 1 month old) were grown under a day and night (10 h light/14 h dark) cycle. Scale bar = 1 cm. (**B**) Leaf surface temperature of plants in (**A**) observed by a thermal imaging camera.

To examine whether the lack of NPF4.6 proteins present in guard cells is the cause of the reduced leaf surface temperature in the *aao3 npf4.6* double mutant compared to the single *aao3* mutant, we expressed wild-type *NPF4.6* using a guard cell-specific Arabidopsis *MYB60* promoter in the double mutant background (Figure 3). Three independent transgenic lines had higher leaf surface temperatures compared to the original *aao3 npf4.6* double mutant, although there were variations in the degree of the effect. Altogether, these observations support the idea that NPF4.6 localized in guard cells facilitates ABA uptake into the cells and promotes stomatal closure.

### 3.2. Identification of Another NPF That Regulates Stomatal Aperture

Our previous study showed that several Arabidopsis NPF proteins other than NPF4.6 were able to induce interactions between the ABA receptor PYR1 and the PP2C protein phosphatase ABI1 in yeast under low ABA concentrations in the growth media [35,46], indicating that other NPF proteins can mediate ABA uptake into the cells. NPF5.1 was one of the identified proteins [46], and transcriptome data available in a public database (Arabidopsis eFP Browser; http://bar.utoronto.ca/efp/cgi-bin/efpWeb.cgi, accessed on 5 April 2016) indicate that the expression of *NPF5.1* is induced by exogenous ABA treatment (Supplemental Figure S1). Thus, we hypothesized that NPF5.1 might somehow be involved in ABA-mediated physiological responses and examined this possibility.

**Figure 4:**
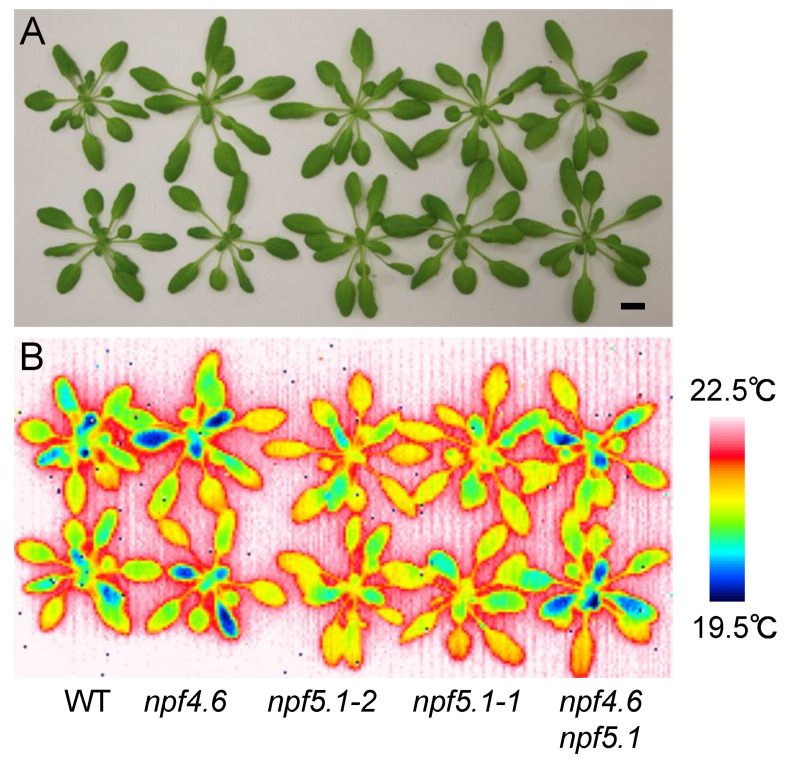
*npf5.1* has a higher leaf surface temperature compared to the wild type. (**A**) Rosette leaves of wild type (WT), *npf4.6-1* (*npf4.6*), *npf5.1-1*, *npf5.1-2* and *npf4.6-1 npf5.1-1* (*npf4.6 npf5.1*) plants (approximately 1 month old) grown under a day and night (10 h light/14 h dark) cycle. Scale bar = 1 cm. (**B**) Leaf surface temperature of plants in (**A**) observed by a thermal imaging camera immediately after detachment of the rosette leaves from the roots.

We obtained two alleles of the loss-of-function *npf5.1* mutants (*npf5.1-1* and *npf5.1-2*) from T-DNA insertion lines (Supplemental Figure S2). Observing the mutants with an infrared thermal imaging camera showed that both mutants had higher leaf surface temperatures compared to the wild type, suggesting that NPF5.1 is a negative regulator of stomatal closure (Figure 4). In this experiment, we took thermographic images just after detaching the rosette leaves from roots. This step was necessary because the phenotype was relatively weak, and the leaf surface temperature was affected by its contact with the wet soil when observations were made on intact potted plants. Again, clear differences in the leaf surface temperature were not observed between wild type and the *npf4.6* single mutant. If, however, we introduced an *npf5.1* mutation into the *npf4.6* mutant background, the phenotype observed in the original *npf5.1* mutant was suppressed (Figure 4). This result supports the idea that NPF4.6 is a positive regulator of stomatal closure. In addition, the higher leaf surface temperature observed in *npf5.1* is likely to require the function of NPF4.6.

### 3.3. NPF5.1 Has an ABA Uptake Activity

**Figure 5:**
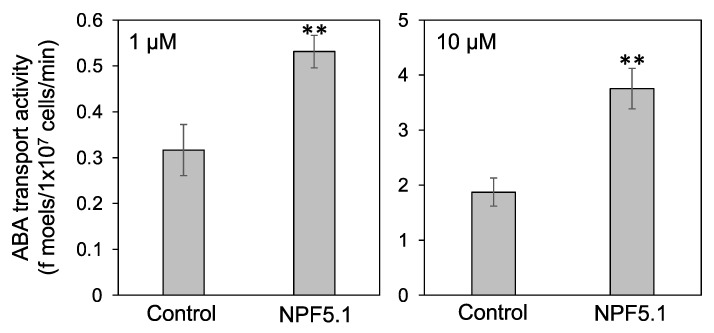
ABA transport activity of NPF5.1. The amounts of ABA (f moels/1 × 10^7^ cells/min) taken into yeast cells expressing NPF5.1 or control cells containing an empty vector were determined when the substrate concentration was 1 or 10 μM. Results are presented as mean ± SD of three biological replicates. ** Significantly different compared with the values in the control cells (*p* < 0.001) by Student’s *t*-test.

We then examined whether NPF5.1 can transport ABA. Yeast cells expressing NPF5.1 were incubated with solutions containing ABA, and the amount of compound taken into the cells was quantified by liquid chromatography-tandem mass spectrometry (LC-MS/MS). The results clearly showed that NPF5.1 mediated cellular ABA uptake when the substrate concentrations were 1 and 10 μM (Figure 5).

**Figure 6:**
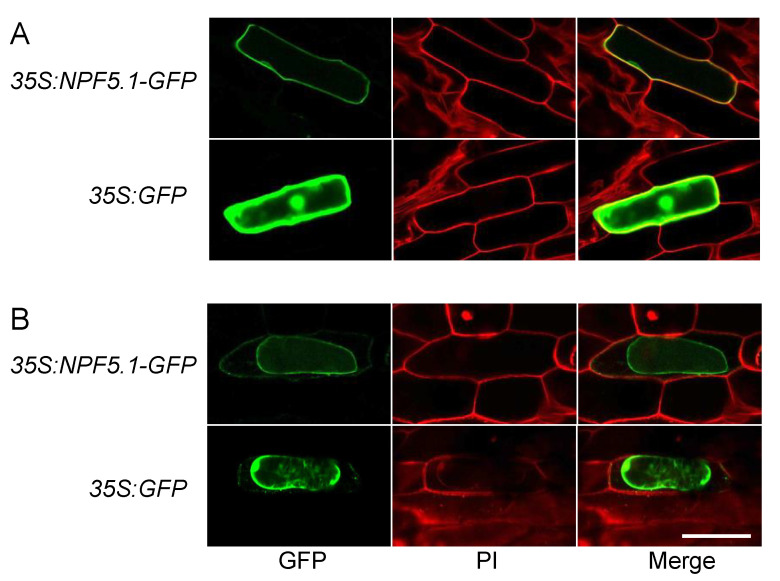
Plasma membrane localization of NPF5.1. GFP signals in onion epidermal cells that transiently express GFP-fused NPF5.1 or GFP alone under the control of the *35S* promoter (*35S:NPF5.1-GFP* and *35S:GFP*, respectively). Photos were taken before (**A**) and after (**B**) plasmolysis with 20% mannitol. PI indicates propidium iodide staining of the cell walls. Scale bar = 200 μm.

The accumulation of ABA in the NPF5.1-expressing yeast cells indicated that the protein was localized to the plasma membrane. To determine if this localization site is also the case in plant cells, we transiently expressed GFP-fused NPF5.1 proteins in onion epidermal cells by particle bombardment (Figure 6). Fluorescence was detected broadly in the cells expressing GFP alone; in contrast, the signals were localized to the plasma membrane in the cells expressing NPF5.1-GFP fusion proteins.

**Figure 7:**
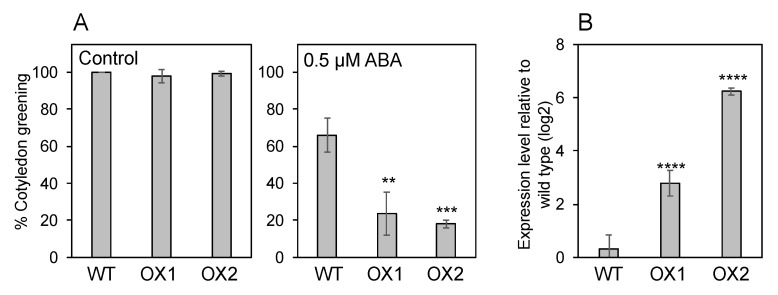
Overexpression of *NPF5.1* enhances ABA sensitivities during germination. (**A**) Cotyledon greening of wild type (WT) and two independent transgenic lines that overexpress *NPF5.1* (OX1 and 2) on control media without supplementation of ABA or media containing 0.5 μM ABA scored at 4 d (control) and 7 d (0.5 μM ABA) after stratification. (**B**) Expression levels of *NPF5.1* in 7-d-old seedlings of wild type (WT) and two independent transgenic lines that overexpress *NPF5.1* (OX1 and 2). Asterisks indicate statistically significant differences compared to the wild type determined by Dunnett’s multiple comparison test (** *p* < 0.01; *** *p* < 0.001; **** *p* < 0.0001).

If NPF5.1 is localized to the plasma membrane and mediates cellular ABA uptake, overexpression of the protein should result in enhanced ABA sensitivity as we have shown for NPF4.6 [35]. Two independent transgenic lines that overexpress *NPF5.1* (*35S:NPF5.1*) were tested for their responsiveness to exogenously applied ABA during germination. Our result indicates that the overexpressors were more sensitive to the treatment in terms of inhibiting cotyledon greening compared to the wild type (Figure 7).

### 3.4. Endogenous ABA Levels in NPF5.1

ABA is reported to move long distances within plants, for example, from roots to shoots and vice versa [31]. Therefore, the increased leaf surface temperature observed in *npf5.1* might be caused by an accumulation of ABA in leaves due to altered long-distance ABA transport. To examine this possibility, we quantified the endogenous ABA content in rosette leaves of wild type and *npf5.1* (Supplemental Figure S3) and found that the levels were comparable between wild type and the mutant.

### 3.5. Spatial Expression Patterns of NPF5.1

**Figure 8:**
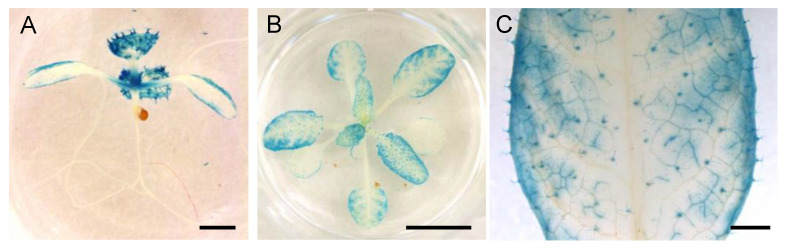
GUS staining of *pNPF5.1:GUS* plants. (**A**) A seedling (approximately 7-d-old) grown under continuous light. Scale bar = 1 mm. (**B**) Rosette leaves of plants grown under continuous light (approximately 3 weeks old). Scale bar = 1 cm. (**C**) Close-up view of a leaf in (**B**). Scale bar = 1 mm.

To understand how NPF5.1 regulates stomatal aperture, it is important to know where the protein functions. We found that promoter activities of *NPF4.6* and NPF4.6-GUS proteins were not always detected in the same cells (Figure 1) [35]. Therefore, we first generated transgenic plants that express GUS-fused NPF5.1 proteins under the control of the *NPF5.1* promoter; however, we were unable to detect GUS activities in the transgenic lines possibly due to low levels of protein accumulation. Accordingly, we visualized promoter activities of *NPF5.1* using a 3 kb region upstream of the ATG start codon and the GUS reporter gene (*pNPF5.1:GUS*) (Figure 8). In young seedlings, the *NPF5.1* promoter was active in true leaves, weakly active in cotyledons, and no activity was detected in the roots. In rosette leaves, GUS staining was observed in many leaf tissues, including mesophyll/epidermal cells, vascular tissues and trichomes. Unlike the case of *NPF4.6*, the promoter activities of *NPF5.1* were not strong in the primary and secondary leaf veins. Furthermore, no GUS activity was specifically detected in the guard cells of the transgenic plants.

## 4. Discussion

Although earlier studies reported that ABA is synthesized in the roots in response to soil drying, it is now widely accepted that drought-inducible ABA biosynthesis takes place mainly in leaf vascular tissues [31]. ABA synthesized in the guard cells has also been reported to induce stomatal closure [55]. The recent identification of ABA transporters indicates that stomatal aperture is regulated by multiple steps through ABA export and import between the vascular tissues and guard cells [10,31]. We previously identified Arabidopsis NPF4.6 as an ABA importer [35]. Promoter-reporter analysis indicated that *NPF4.6* was expressed in leaf vascular tissues and inflorescence stems. Therefore, we hypothesized that NPF4.6 limits the amount of ABA exported from the vascular tissues by facilitating cellular ABA uptake at the site of its active biosynthesis. However, this model cannot explain completely the reduced stomatal apertures observed in the inflorescence stems of *npf4.6*. In addition, the contribution of NPF4.6 in regulating stomatal aperture in leaves was not clear. In this study, we found that the *aao3 npf4.6* double mutant had a lower leaf surface temperature compared to the *aao3* single mutant (Figure 1), indicating that NPF4.6 is potentially a positive regulator of stomatal closure in leaves. Again, this finding is somewhat contradictory to the possible function of NPF4.6 as an ABA importer that is localized in leaf vascular tissues; one may expect that the loss of NPF4.6 function would result in an increased amount of ABA translocated to guard cells and, hence, reduced stomatal aperture. One possibility to explain these results would be that NPF4.6 is present in guard cells, although promoter activity of *NPF4.6* was not detected there. In fact, GUS activities were detected in guard cells as well as in vascular tissues in leaves of *pNPF4.6:gNPF4.6-GUS* transgenic plants (Figure 2). Regulatory regions for transcription and/or translation may be present in the *NPF4.6* genome sequence. It is also possible that NPF4.6 proteins are stabilized by unknown factors in guard cells. Expression of *NPF4.6* specifically in the guard cells of *aao3 npf4.6* increased the leaf surface temperature of the double mutant (Figure 3), indicating that the positive effect of NPF4.6 on stomatal closure relies on the proteins localized in guard cells. Variations in the effects of guard cell-expressed *NPF4.6* on leaf surface temperature among the three independent lines tested in this study are possibly due to different expression levels of the transgene that can be influenced by the location of the insertion sites and/or copy number. The physiological roles of NPF4.6 present in vascular tissues are still unknown. In the future, it will be important to precisely determine the localization of NPF4.6 proteins within vascular tissues and to see the effects of cell type-specific expression of NPF4.6 on the phenotypes of *aao3 npf4.6*.

NPF proteins were originally identified as nitrate or peptide transporters [56]. More recent studies have demonstrated that the member proteins of this family transport a variety of compounds, including plant hormones (ABA, gibberellin (GA), auxin and jasmonoyl isoleucine (JA-Ile)) [35,57,58,59,60,61], secondary metabolites (glucosinolates and alkaloids) [62,63,64], and nutrients (potassium and chloride) [65,66,67]. The Arabidopsis genome encodes 53 NPFs; however, their biochemical and physiological functions are still largely unknown [68]. We previously identified several NPFs that can transport ABA [35,46]. Among them, NPF5.1 had a relatively weak impact on the ABA-dependent PYR1–ABI1 interactions in yeast [46], but we confirmed that the protein did mediate ABA uptake into the cells by direct analysis with LC-MS/MS (Figure 5). The apparently low ABA transport activities of NPF5.1 might be due to lower protein accumulation levels in yeast, although we could not determine this possibility. Thus, it is still possible that NPF5.1 could efficiently transport ABA within plants. Our previous study also indicated that NPF5.1 transports GA in addition to ABA when expressed in yeast [46]. We hypothesize that NPF5.1 is a multifunctional transporter as has been reported, for example, for NPF4.6 (nitrate and ABA) [35,69], NPF2.10 (glucosinolates, GA and JA-Ile) [58,61,62], and NPF7.3 (nitrate, potassium and indole-3-butyric acid) [52,67,70]. Nevertheless, we focused on the role of NPF5.1 on ABA transport in this study.

The expression of genes involved in ABA biosynthesis, catabolism, signaling and transport are reportedly regulated by the hormone. The Arabidopsis eFP Browser (http://bar.utoronto.ca/efp/cgi-bin/efpWeb.cgi, accessed on 5 April 2016) indicated that the expression of *NPF5.1* is induced by exogenous ABA treatment ( Supplemental Figure S1). Therefore, we hypothesized that NPF5.1 might also be involved in ABA-mediated physiological responses, although the physiological meaning of this gene expression was not known. Although NPF4.6 positively regulates stomatal closure, NPF5.1 appeared to function as a negative regulator of the process because the loss-of-function *npf5.1* mutants had higher leaf surface temperatures compared to the wild type (Figure 4). Based on the observation that the promoter activities of NPF5.1 were detected in leaf vascular tissues as well as in the mesophyll and/or epidermal cells (Figure 8), we propose a model in which NPF5.1 regulates the amount of ABA transported from the vascular tissues to the guard cells by mediating cellular ABA uptake (Supplemental Figure S4). As mentioned above, promoter activity and protein accumulation can be detected in different places, but we were unable to observe the distribution of NPF5.1. Tissue/cell-type-specific expression of *NPF5.1* in the *npf5.1* mutant background and monitoring the effects on the mutant phenotype will be required to identify the functional sites of NPF5.1. According to our model, the loss-of-function of both NPF4.6 and NPF5.1 should result in an increased amount of ABA that can travel through the apoplastic space. Nevertheless, *npf4.6 npf5.1* had lower leaf surface temperatures compared to *npf5.1*. This result can be explained by the defect in NPF4.6 that mediates ABA uptake into the guard cells (Supplemental Figure S4).

## 5. Conclusions

In conclusion, our study demonstrates that two Arabidopsis NPF proteins, which function as ABA importers, positively and negatively regulate leaf stomatal closure by mediating ABA uptake in different tissues and cells.

## Data Availability

All the data supporting the results of this paper are presented in the paper and/or the supplementary materials.

## Supplementary Materials

The following are available online at https://www.mdpi.com/article/10.3390/genes12060885/s1, Figure S1: ABA-inducible expression of *NPF5.1*, Figure S2: Genome structure of *NPF5.1* and T-DNA insertion sites of *npf5.1-1* and *npf5.1-2*, Figure S3: Endogenous ABA levels in wild type and *npf5.1*, Figure S4: A model showing the possible functions of NPF4.6 and NPF5.1 in vascular tissues, leaf mesophyll and epidermal cells and/or guard cells.
