# Supplementary material for: Arabidopsis NPF4.6 and NPF5.1 Control Leaf Stomatal Aperture by Regulating Abscisic Acid Transport
Source: Genes (Basel). 2021 Jun 8;12(6):885. doi: 10.3390/genes12060885 (PMC8227765; doi:10.3390/genes12060885)
Supplement: Supplementary file 1 [file genes-12-00885-s001.zip › genes-1232363-supplementary.pdf]

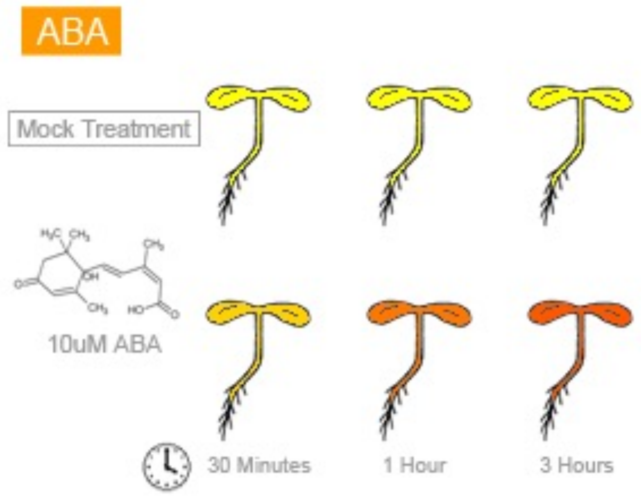

Figure S1. ABA inducible expression of *NPF5.1*. The image was obtained from Arabidopsis eFP Browser (http://bar.utoronto.ca/efp/cgi-bin/efpWeb.cgi).

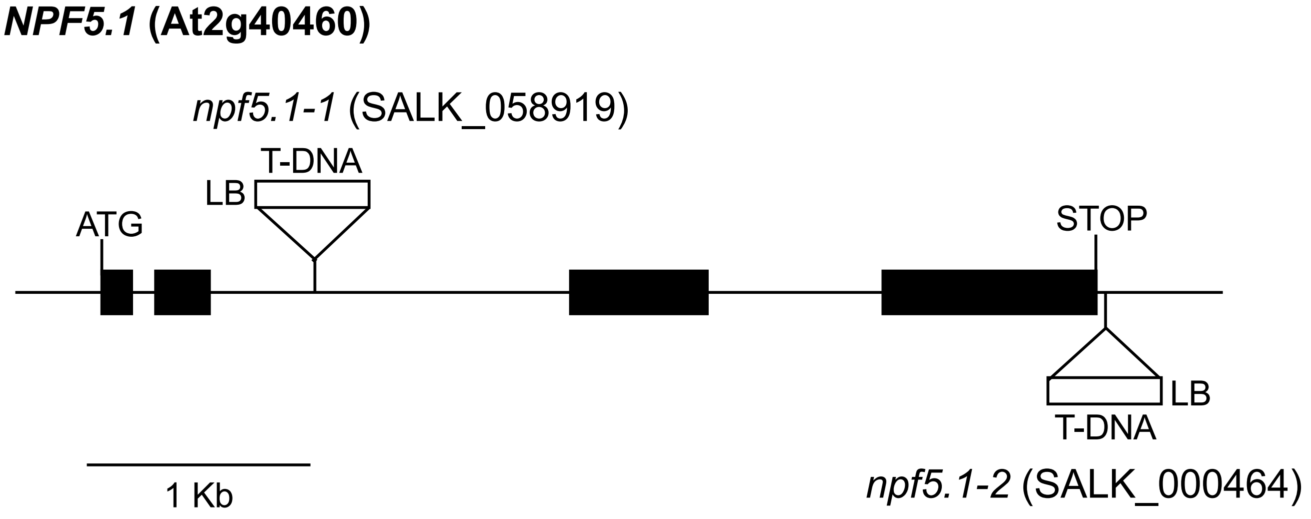

Figure S2. Genome structure of *NPF5.1* and T-DNA insertion sites for *npf5.1-1* and *npf5.1-2*.

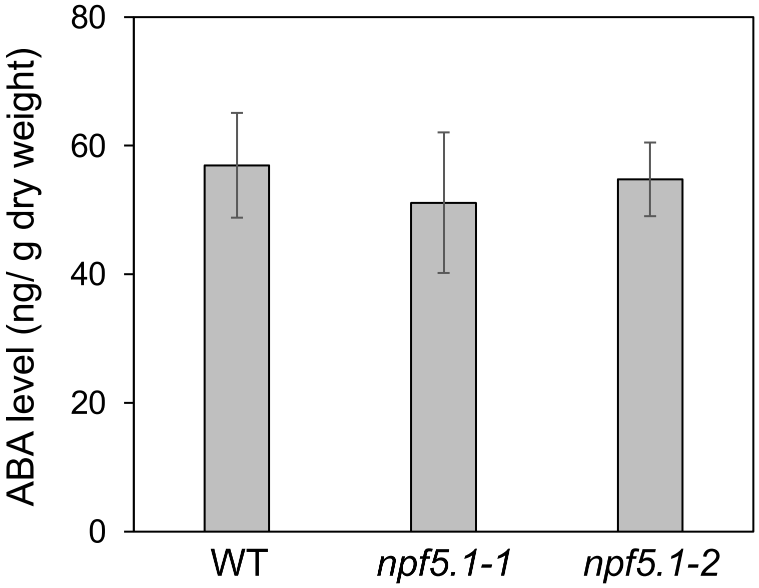

Figure S3. Endogenous ABA levels in wild type and *npf5.1*. ABA levels were quantified from one whole rosettes (around 1 month old) and the results were shown as means ± SD of 8 (wild type and *npf5.1-1*) or 7 (*npf5.1-2*) independent samples. Significant difference in the values was not detected among the genotypes by Tukey's multiple comparison test.

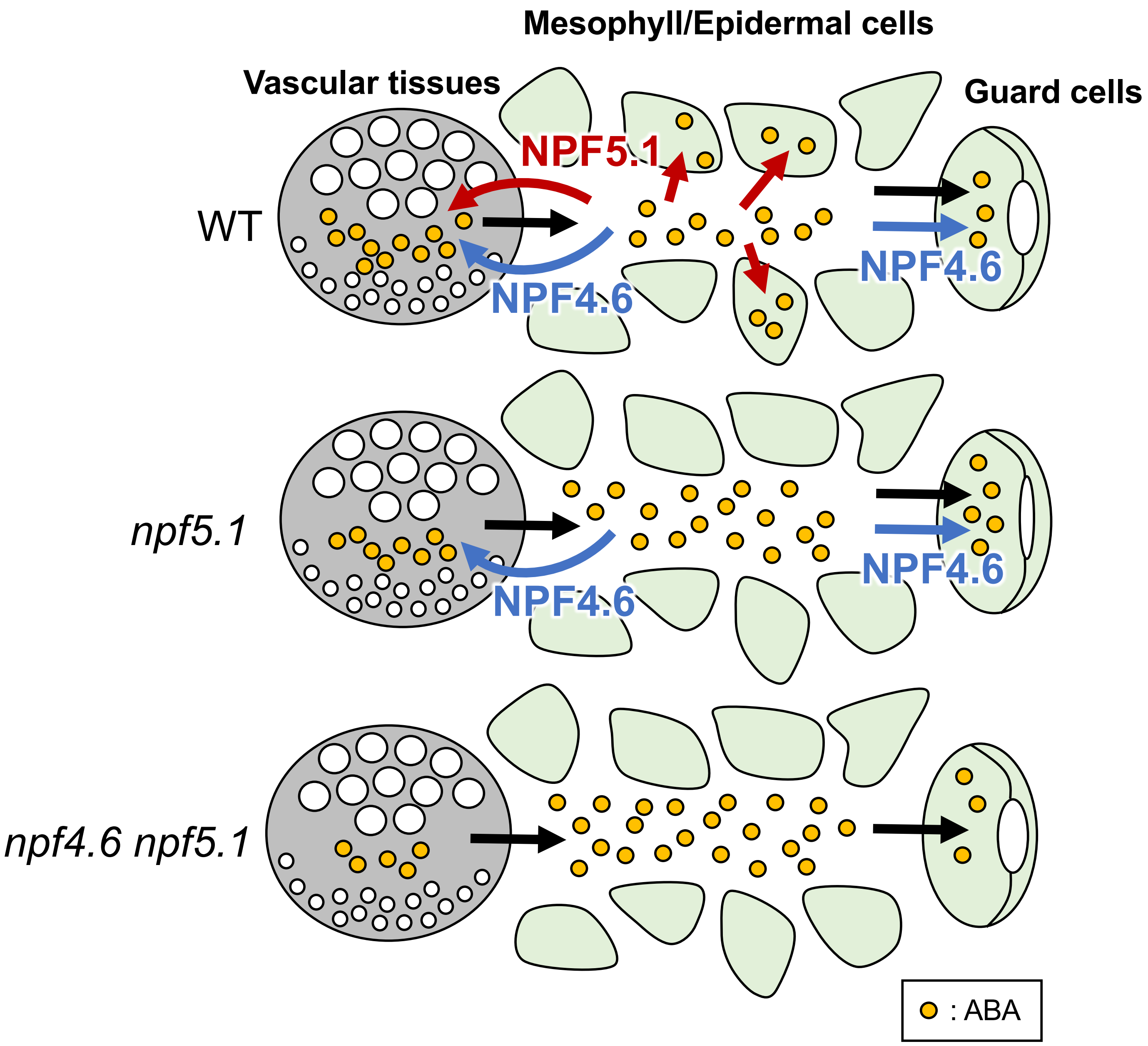

Figure S4. A model showing the possible functions of NPF4.6 and NPF5.1. Blue and red arrows indicate cellular ABA uptake mediated respectively by NPF4.6 and NPF5.1 in vascular tissues, leaf mesophyll and epidermal cells and/or guard cells. Black allows indicate ABA transport mediated by other ABA transporters.
